# Intratumour heterogeneity in the uptake of macromolecular therapeutic agents in human melanoma xenografts

**DOI:** 10.1038/sj.bjc.6600680

**Published:** 2003-01-28

**Authors:** B A Graff, Y Kvinnsland, A Skretting, E K Rofstad

**Affiliations:** 1 Department of Biophysics, Institute for Cancer Research, The Norwegian Radium Hospital, Montebello, N-0310 Oslo, Norway; 2 Department of Medical Physics, The Norwegian Radium Hospital, Montebello, N-0310 Oslo, Norway

**Keywords:** macromolecule uptake, melanoma, microvasculature, interstitium, blood supply, perfusion, transport mechanisms

## Abstract

Intratumour heterogeneity in the uptake of blood-borne technetium-labelled human serum albumin (^99m^Tc-HSA) was studied in human melanoma xenografts in an attempt to identify transport barriers leading to inadequate and heterogeneous uptake of macromolecular therapeutic agents in tumours. The Bioscope imaging system, which can detect the distribution of ^99m^Tc in 10-*μ*m-thick tissue sections with a spatial resolution of just above 50 *μ*m, was used to image the ^99m^Tc-HSA uptake. Xenografted tumours of four human melanoma cell lines were included in the study. Significant intratumour heterogeneity in the uptake of ^99m^Tc-HSA was detected. The heterogeneity had two distinctly different components, one random and one radial component. The uptake was lowest in the centre of the tumours and increased towards the tumour periphery. This radial heterogeneity was superimposed by a random heterogeneity, that is, spots with high uptake colocalised with spots with high vascular density and regions without significant uptake colocalised with necrotic regions. The magnitude of the heterogeneity did not change significantly with time after the administration of ^99m^Tc-HSA. The tumours showed a random and a radial heterogeneity in blood perfusion similar to that in the uptake of ^99m^Tc-HSA. The observations reported here suggest that the intratumour heterogeneity in the distribution of ^99m^Tc-HSA was initiated primarily because of heterogeneity in the supply of ^99m^Tc-HSA through the microvasculature, and that the presence of severe transport barriers in the tumour interstitium prevented significant equalisation of the initial heterogeneity with time. Consequently, strategies for improving the delivery of macromolecular therapeutic agents to tumours should focus on increasing the tumour blood perfusion to increase the total uptake and improving the diffusion conditions in the tumour interstitium to diminish the heterogeneity in the uptake.

Novel strategies for the treatment of cancer involving macromolecular therapeutic agents such as growth factors, monoclonal antibodies, immunomodulators, oligonucleotides, gene therapy vectors and encapsulated drugs have been developed recently (Jain, 1998). Preclinical studies of the therapeutic usefulness of such agents in several *in vitro* and *in vivo* cancer models have given promising results (Yuan, 1998). However, inadequate and heterogeneous uptake in tumour tissue has been shown to be a major obstacle for efficient use of macromolecules in clinical cancer therapy (Yuan, 1998; Jain, 2001). Improved strategies are therefore needed to increase the macromolecule delivery to tumours.

The uptake of macromolecular therapeutic agents in tumour tissue depends on the physical and chemical properties of the macro-molecule and the biology of the tissue. Macromolecular properties exerting a significant influence on the uptake include molecular weight, shape, charge and affinity for binding to stromal and parenchymal tissues (Dellian *et al*, 2000). Biological factors that may prevent adequate uptake include low microvascular density, poor perfusion, low transvascular permeability, high interstitial fluid pressure and a dense, well-organised extracellular matrix (Hobbs *et al*, 1998). However, the mechanisms regulating the uptake are not well understood. Novel strategies for increasing the delivery can probably not be developed without identification of the barriers limiting the uptake. Such identification requires care-ful studies with well-characterised experimental tumours and macro-molecules with well-known physical and chemical properties.

The mechanisms governing the uptake of macromolecular therapeutic agents in human melanoma xenografts are currently being investigated in our laboratory, using albumin as a model molecule for macromolecular therapeutic agents (Graff *et al*, 2000, 2001; Bjørnæs and Rofstad, 2001). We have previously measured the global uptake of blood-borne albumin, and found that it was strongly correlated to the extracellular volume fraction of the tumour tissue (Graff *et al*, 2000) and was not limited by the permeability of the microvascular wall (Bjørnæs and Rofstad, 2001; Graff *et al*, 2001). In the present work, the intratumour heterogeneity in the uptake of technetium-labelled human serum albumin (^99m^Tc-HSA) was studied and related to the intratumour heterogeneity in necrosis, vascular density, blood perfusion and extracellular volume fraction. Human melanoma xenografts having retained essential biological features of the donor patients’ tumours, including histology, growth rate and vascularisation, have been established and characterised in our laboratory (Rofstad, 1994). These xenografts were used here, and the studies demonstrated time-independent intratumour heterogeneity in ^99m^Tc-HSA uptake, caused primarily by heterogeneous supply of ^99m^Tc-HSA through the tumour microvasculature and poor diffusion conditions for ^99m^Tc-HSA in the tumour interstitium.

## Materials and methods

### Mice and tumours

Adult (8−12 weeks of age) female BALB/c-*nu/nu* mice, bred at our research institute, were used as host animals for xenografted tumours. The mice were maintained under specific pathogen-free conditions at constant temperature (24−26°C) and humidity (30−50%). Sterilised food and tap water were given *ad libitum.* Four human melanoma lines (A-07, D-12, R-18 and U-25), described in detail elsewhere (Rofstad, 1994), were included in the study. Tumours were initiated from exponentially growing monolayer cultures verified to be free from *Mycoplasma* contamination. The cells were cultured in RPMI-1640 medium (25 mM HEPES and L-glutamine) supplemented with 13% bovine calf serum, 250 mg l^−1^ penicillin and 50 mg l^−1^ streptomycin. Approximately 3.5×10^5^ cells in 10 *μ*l of Ca^2+^- and Mg^2+^-free Hanks’ balanced salt solution were inoculated intradermally into the flanks of mice by using a 100-*μ*l Hamilton syringe. The tumours were deposited above the subcutaneous muscle tissue in the deeper part of the dermis. The growth and histological appearance of the tumours have been described elsewhere (Rofstad, 1994). D-12 and U-25 tumours develop necrotic regions during growth, whereas A-07 and R-18 tumours do not. Tumours with volumes within the range of 300−600 mm^3^ were subjected to investigation. Tumour volume (*V*) was calculated as *V*=(π/6) *ab*^2^, where *a* is the longer and *b* is the shorter of two perpendicular diameters, measured with callipers. Animal experiments were approved by the Institutional Committee on Research Animal Care and were performed according to the ethical standards of the UKCCCR ‘Guidelines for the Welfare of Animals in Experimental Neoplasia’ (Workman *et al*, 1998).

### Bioscope

**Figure 1:**
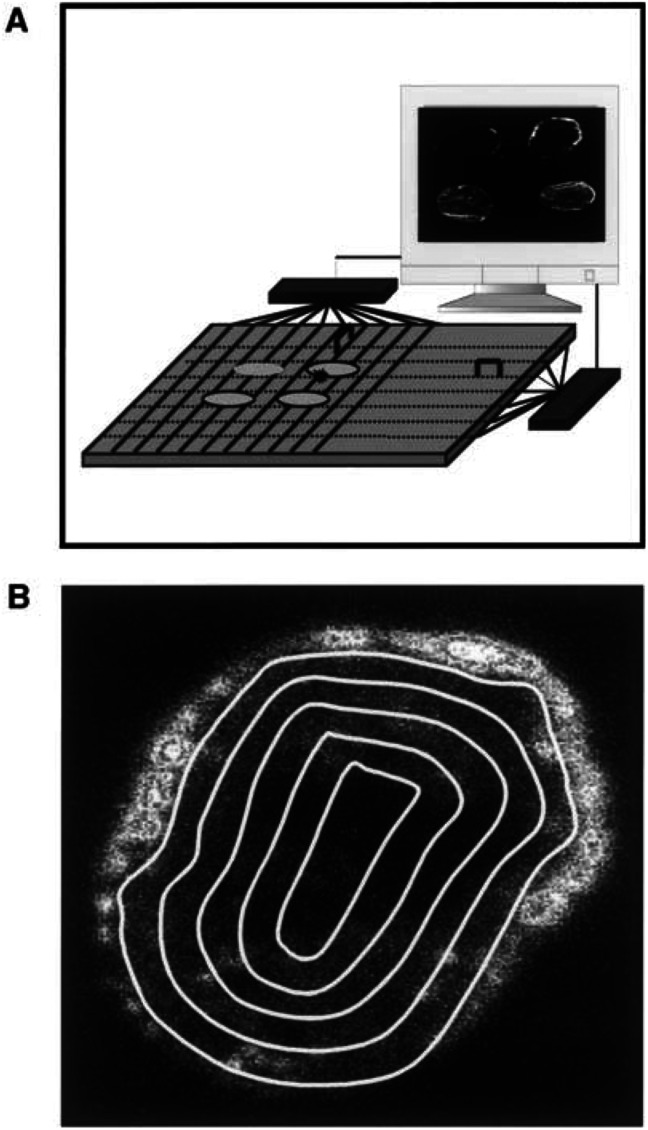
(**A**) Schematic illustration of the Bioscope system. The sensor of the system is a 300-*μ*m-thick double-sided silicon strip detector with a sensitive area of 32×32 mm. The detector has 640 strips in each direction. Four tumour slices can be imaged simultaneously. (**B**) Bioscope image of an A-07 tumour. The concentration of ^99m^Tc in the tumour tissue is proportional to the intensity in the image. Quantitative analysis of the radial distribution of ^99m^Tc was performed by dividing the images into five sectors and analysing each sector separately. The sectors were bounded by lines drawn at distances of *nR*/5 from the tumour centre, where *R* is the tumour radius and *n* is the sector number.

The Bioscope system (IDE AS, Oslo, Norway), designed to image the distribution of isotopes in tissue sections with high spatial resolution (Overdick *et al*, 1997), was used to study intratumour heterogeneity in macromolecule uptake, extracellular volume fraction and blood perfusion (Figure 1

). The sensor of this system is a 300-*μ*m-thick double-sided silicon strip detector with a sensitive area of 32×32 mm. The detector has 640 strips in each direction, that is, a strip pitch of 50 *μ*m. Four tumour sections can be imaged simultaneously, as illustrated in Figure 1A. The positions of ionising events are registered by the detector and transferred to a computer. Low-energy *β*-emitters, such as ^99m^Tc, give images with a spatial resolution of just above 50 *μ*m. Quantitative analysis of images was performed by using the analySIS image processing system (Soft Imaging System GmbH, Münster, Germany). The radial distribution of activity was determined by dividing the tumour images into five sectors and analysing each sector separately, as illustrated in Figure 1B. The sectors were bounded by lines drawn at distances of *nR*/5 from the tumour centre, where *R* is the tumour radius and *n* is the sector number. The skin surrounding the tumours was excluded from the analyses.

### Macromolecule uptake

^99m^Tc-HSA was diluted in 0.9% saline and administered intra-venously to tumour-bearing mice in a bolus dose of 32 mg kg^−1^ (approximately 8×10^2^ MBq kg^−1^). The mice were killed by cervical dislocation 1, 5, 10, 30, 60, 120 or 180 min after the administration, and the tumours were frozen in liquid nitrogen and cut into two halves. One of the halves was subjected to measurement of activity in a gamma counter (Wallac Oy, Turku, Finland) to ascertain that the kinetics of the global uptake was consistent with previous measurements. Frozen sections of 10-*μ*m thickness were prepared from the other halves, using standard procedures. The spatial distribution of activity in these sections, that is, the concentration of ^99m^Tc-HSA, was imaged with the Bioscope system and used as a parameter for the intratumour heterogeneity in macromolecule uptake. The sections were stained with haematoxylin and eosin after the imaging and examined by light microscopy.

### Extracellular volume fraction

^99m^Tc-HSA in doses similar to those described above was administered intravenously to tumour-bearing mice with ligated renal arteries. The procedure used for the ligation of renal arteries has been described elsewhere (Bjørnæs *et al*, 2000). The mice were killed and the tumours were frozen in liquid nitrogen 3 h after the administration of ^99m^Tc-HSA. Frozen sections (10-*μ*m thick) were prepared from the tumours, and the spatial distribution of activity in the sections, that is, the concentration of ^99m^Tc-HSA, was imaged with the Bioscope system and used as a parameter for the intratumour heterogeneity in extracellular volume fraction.

### Blood perfusion

Na^99m^TcO_4_ was administered intravenously to tumour-bearing mice in a bolus dose (approximately 8×10^2^ MBq kg^−1^). The mice were killed and the tumours were frozen in liquid nitrogen 45 s after the administration of Na^99m^TcO_4_. Frozen sections (10-*μ*m thick) were prepared from the tumours, and the spatial distribution of activity in the sections, that is, the concentration of Na^99m^TcO_4_, was imaged with the Bioscope system and used as a parameter for the intratumour heterogeneity in perfusion.

### Statistical analysis

Results are presented as arithmetic mean±s.e.m. The Pearson Product Moment Correlation test was used under conditions of normality and equal variance to determine the strength of correlations between variables. The statistical analysis was performed by using SigmaStat statistical software (Jandel Scientific GmbH, Erkrath, Germany).

## Results

### Qualitative studies

Qualitative examinations of Bioscope images revealed significant intratumour heterogeneity in the uptake of ^99m^Tc-HSA. The heterogeneity had two distinctly different components, one random and one nonrandom radial component. The uptake was lowest in the centre of the tumours and increased towards the tumour periphery. This radial heterogeneity was superimposed by a random heterogeneity. A-07 and R-18 tumours showed spots with high uptake and randomly distributed within the tissue. D-12 and U-25 tumours showed randomly distributed regions without significant uptake in addition to spots with high uptake.

**Figure 2:**
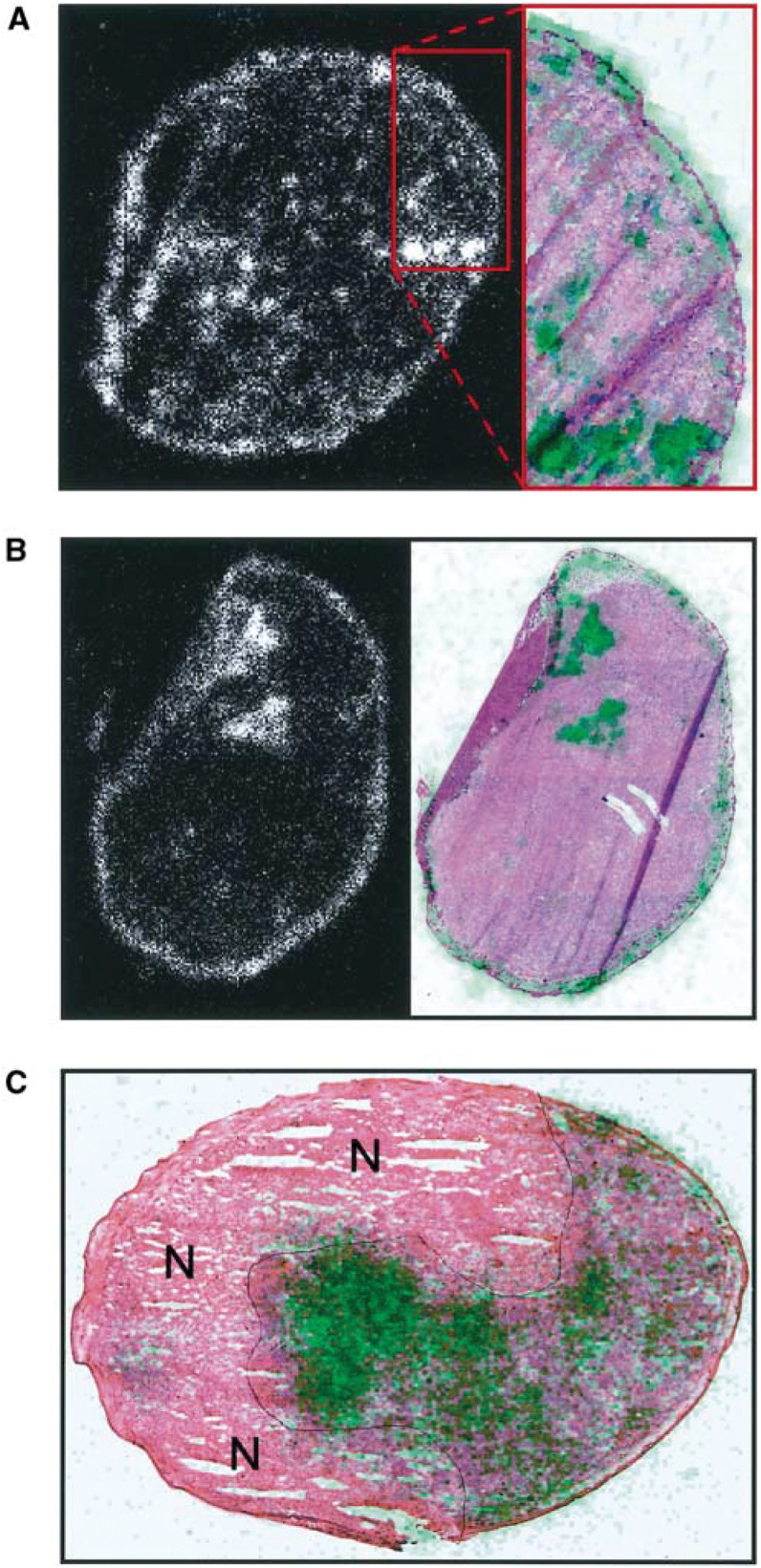
(**A**) Left: Bioscope image of an R-18 tumour. ^99m^Tc-HSA was administered intravenously to the tumour-bearing mouse. The mouse was killed and the tumour was frozen in liquid nitrogen 1 min after the administration of ^99m^Tc-HSA. The concentration of ^99m^Tc-HSA in the tumour tissue is proportional to the intensity in the Bioscope image. Right: histological image of the tumour region demarcated with red lines in the Bioscope image. Low-pass-filtered Bioscope image, showing the concentration of ^99m^Tc-HSA in green, is superimposed on the histological image. (**B**) Left: Bioscope image of an R-18 tumour. ^99m^Tc-HSA was administered intravenously to the tumour-bearing mouse. The mouse was killed and the tumour was frozen in liquid nitrogen 60 min after the administration of ^99m^Tc-HSA. The concentration of ^99m^Tc-HSA in the tumour tissue is proportional to the intensity in the Bioscope image. Right: histological image of the same tumour section. Low-pass-filtered Bioscope image, showing the concentration of ^99m^Tc-HSA in green, is superimposed on the histological image. (**C**) Histological image of a D-12 tumour. Low-pass-filtered Bioscope image, showing the concentration of ^99m^Tc-HSA in green, is superimposed on the histological image. ^99m^Tc-HSA was administered intravenously to the tumour-bearing mouse. The mouse was killed and the tumour was frozen in liquid nitrogen 180 min after the administration of ^99m^Tc-HSA. Necrotic regions are indicated by N.

Bioscope images of ^99m^Tc-HSA activity were superimposed on histological images in an attempt to identify the causes of the random component of the heterogeneity (Figure 2

). Spots with high uptake colocalised with spots with high vascular density, either the tumour host was killed minutes (Figure 2A) or hours (Figure 2B) after the administration of ^99m^Tc-HSA. Regions without significant uptake colocalised with necrotic regions independent of the time between the administration of ^99m^Tc-HSA and the killing of the tumour host (Figure 2C).

Moreover, qualitative examinations of Bioscope images of Na^99m^TcO_4_ activity revealed an intratumour heterogeneity in blood perfusion similar to that in the uptake of ^99m^Tc-HSA. The perfusion was lowest in the centre of the tumours and increased towards the tumour periphery. Spots with high perfusion colocalised with spots with high vascular density and regions without perfusion co-localised with necrotic regions (data not shown).

### Quantitative studies

**Figure 3:**
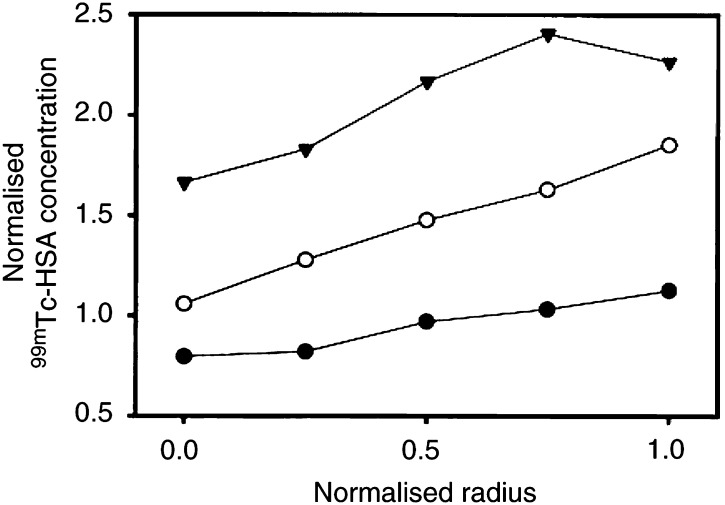
Concentration of ^99m^Tc-HSA vs distance from tumour centre in three individual A-07 tumours. ^99m^Tc-HSA was administered intravenously to tumour-bearing mice. The mice were killed and the tumours were frozen in liquid nitrogen 1 min (•), 30 min (○) or 180 min (▾) after the administration of ^99m^Tc-HSA. Tumour size was normalised, that is, tumour radius was assigned a value of 1.0. The concentration of ^99m^Tc-HSA was also normalised, that is, the mean concentration of ^99m^Tc-HSA in the section from the tumour frozen 1 min after the administration of ^99m^Tc-HSA was assigned a value of 1.0. One section was analysed for each tumour.

The Bioscope images were divided into five sectors (Figure 1B) to quantify the radial heterogeneity in the uptake of ^99m^Tc-HSA. Representative data for A-07 tumours frozen 1, 30 or 180 min after the administration of ^99m^Tc-HSA are presented in Figure 3

, illustrating three general observations: (a) the concentration of ^99m^Tc-HSA increased with time, (b) the concentration of ^99m^Tc-HSA increased linearly with the distance from the tumour centre and (c) the ratio of the concentration of ^99m^Tc-HSA in the tumour periphery and the tumour centre was independent of time within the time interval of 1−180 min. Similar analysis revealed that these three observations were also valid for R-18 tumours (data not shown). Consequently, the magnitude of the radial heterogeneity in the uptake of ^99m^Tc-HSA was independent of time within the time interval of 1−180 min in both A-07 and R-18 tumours, that is, tumours without necrotic regions. Moreover, the uptake of ^99m^Tc-HSA and the magnitude of the radial heterogeneity in the uptake were independent of tumor volume within the volume range studied here, that is, 300–600 mm^3^, in both A-07 and R-18 tumors (data not shown).

**Figure 4:**
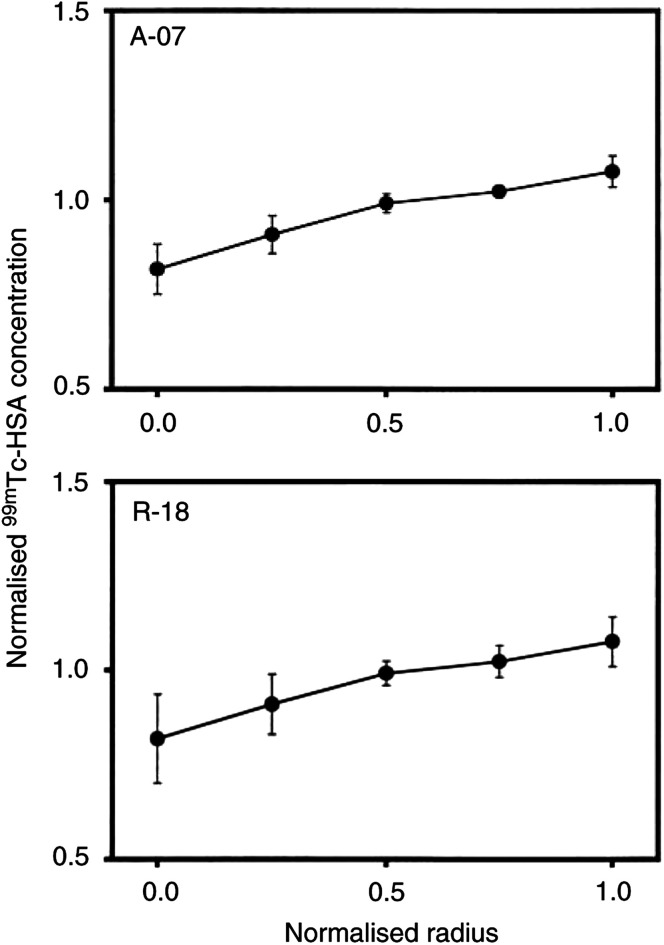
Concentration of ^99m^Tc-HSA *vs* distance from tumour centre in tumours without necrotic regions (A-07 and R-18). ^99m^Tc-HSA was administered intravenously to tumour-bearing mice. The mice were killed and the tumours were frozen in liquid nitrogen 1−180 min after the administration of ^99m^Tc-HSA. Tumour size was normalised, that is, tumour radius was assigned a value of 1.0. The concentration of ^99m^Tc-HSA was also normalised, that is, the mean concentration of ^99m^Tc-HSA in each tumour section was assigned a value of 1.0. One section was analysed for each tumour. Points represent mean values of 11 (A-07) or 7 (R-18) tumours. Bars represent s.e.m.

**Figure 5:**
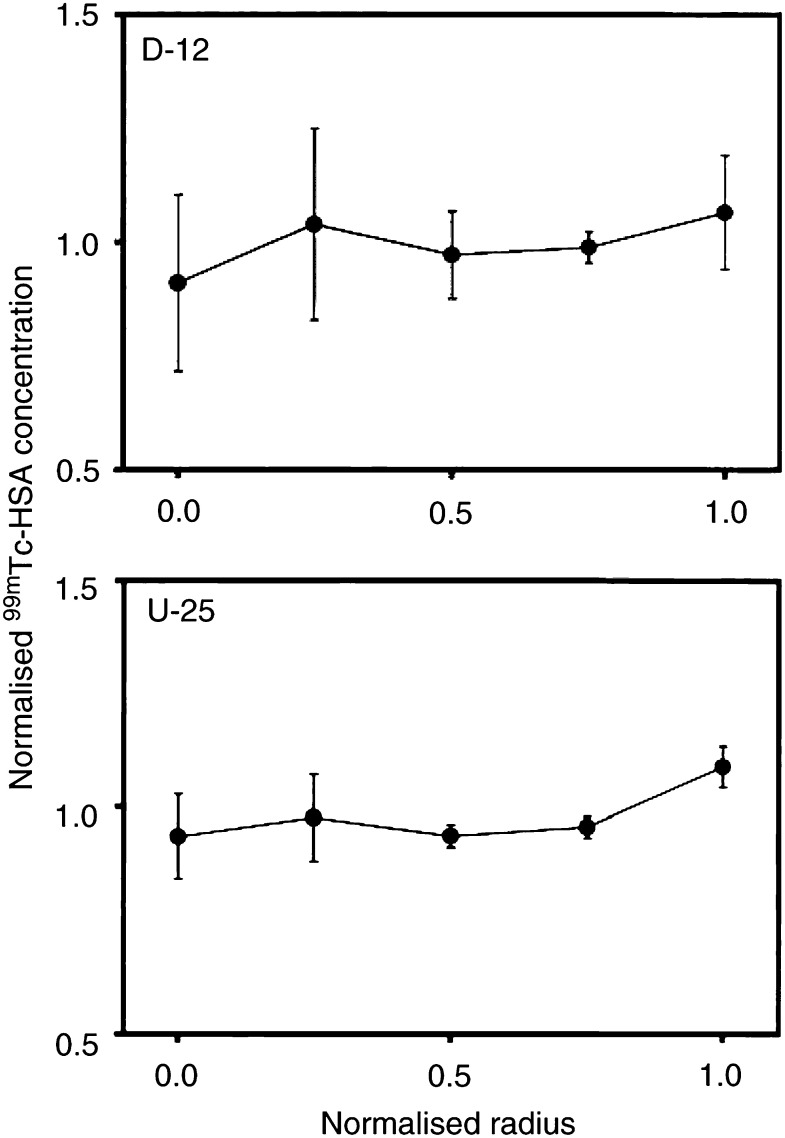
Concentration of ^99m^Tc-HSA *vs* distance from tumour centre in tumours with necrotic regions (D-12 and U-25). ^99m^Tc-HSA was administered intravenously to tumour-bearing mice. The mice were killed and the tumours were frozen in liquid nitrogen 1−180 min after the administration of ^99m^Tc-HSA. Tumour size was normalised, that is, tumour radius was assigned a value of 1.0. The concentration of ^99m^Tc-HSA was also normalised, that is, the mean concentration of ^99m^Tc-HSA in each tumour section was assigned a value of 1.0. One section was analysed for each tumour. Points represent mean values of eight (D-12) or four (U-25) tumours. Bars represent s.e.m.

Data for individual tumours were normalised and pooled to compare the radial heterogeneity in the uptake of ^99m^Tc-HSA in A-07 and R-18 tumours (Figure 4

). A significant difference between the two tumour types could not be detected; the concentration of ^99m^Tc-HSA in the tumour periphery was higher than that in the tumour centre by a factor of approximately 1.5 in both tumour lines (*P*≪0.05). The same procedure was used to quantify the radial heterogeneity in the uptake of ^99m^Tc-HSA in D-12 and U-25 tumours, that is, tumours showing significant necrotic regions (Figure 5

). The quantitative analysis did not reveal a significant radial gradient in the uptake of ^99m^Tc-HSA in these tumour lines (*P*>0.05), although the qualitative examination showed that the uptake in non-necrotic tissue was substantially higher in the tumour periphery than in the tumour centre. Consequently, the data in Figure 5 most likely reflect that necrotic regions without significant uptake of ^99m^Tc-HSA had a random radial distribution and were sufficiently large to govern the overall radial heterogeneity in the uptake of ^99m^Tc-HSA.

**Figure 6:**
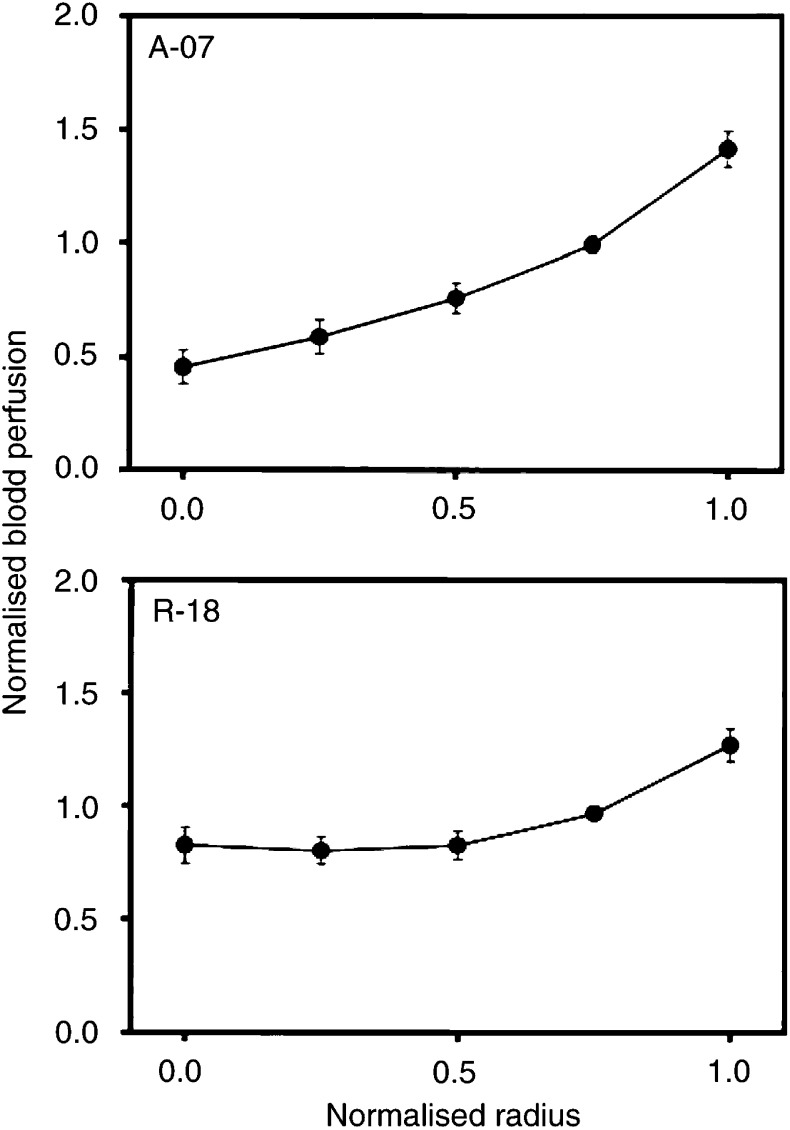
Blood perfusion *vs* distance from tumour centre in A-07 and R-18 tumours. Na^99m^TcO_4_ was administered intravenously to tumour-bearing mice. The mice were killed and the tumours were frozen in liquid nitrogen 45 s after the administration of Na^99m^TcO_4_. The concentration of Na^99m^TcO_4_ in the tumour tissue was used as a parameter for blood perfusion. Tumour size was normalised, that is, tumour radius was assigned a value of 1.0. Blood perfusion was also normalised, that is, the mean concentration of Na^99m^TcO_4_ in each tumour section was assigned a value of 1.0. One section was analysed for each tumour. Points represent mean values of 8 (A-07) or 7 (R-18) tumours. Bars represent s.e.m.

**Figure 7:**
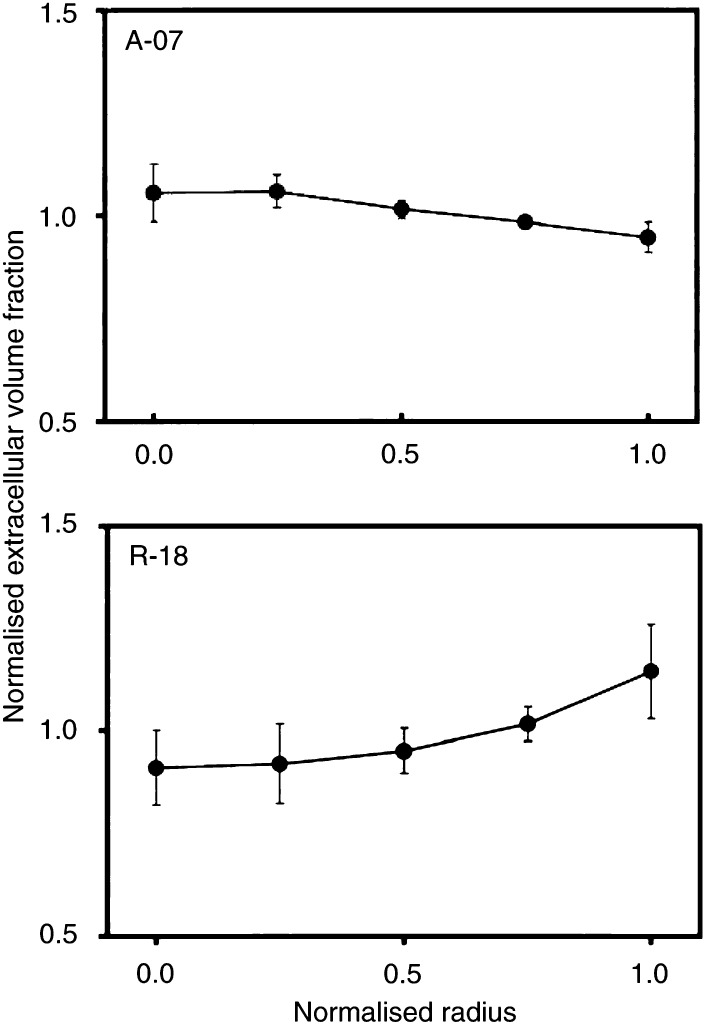
Extracellular volume fraction *vs* distance from tumour centre in A-07 and R-18 tumours. ^99m^Tc-HSA was administered intravenously to tumour-bearing mice with ligated renal arteries. The mice were killed and the tumours were frozen in liquid nitrogen 3 h after the administration of ^99m^Tc-HSA. The concentration of ^99m^Tc-HSA in the tumour tissue was used as a parameter for extracellular volume fraction. Tumour size was normalised, that is, tumour radius was assigned a value of 1.0. Extracellular volume fraction was also normalised, that is, the mean concentration of ^99m^Tc-HSA in each tumour section was assigned a value of 1.0. One section was analysed for each tumour. Points represent mean values of 8 (A-07) or 7 (R-18) tumours. Bars represent s.e.m.

The radial distributions in blood perfusion (Figure 6

) and extracellular volume fraction (Figure 7

) were quantified in A-07 and R-18 tumours in an attempt to identify causes of the radial heterogeneity in the uptake of ^99m^Tc-HSA. The perfusion was lowest in the centre of the tumours and increased towards the tumour periphery. A-07 tumours showed approximately three-fold higher perfusion in the periphery than in the centre (*P*≪0.05), whereas in R-18 tumours, the perfusion in the periphery was higher than that in the centre by a factor of approximately 1.5 (*P*≪0.05). The radial heterogeneity in extracellular volume fraction, on the other hand, was weak in both tumour lines. The extracellular volume fraction decreased slightly towards the tumour periphery in A-07 tumours (*P*=0.030) and increased slightly with the distance from the tumour centre in R-18 tumours (*P*=0.035).

## Discussion

Intratumour heterogeneity in the uptake of ^99m^Tc-HSA was studied in human melanoma xenografts by utilising the unique properties of the newly developed Bioscope imaging system (Overdick *et al*, 1997). This system made it feasible to image the activity of ^99m^Tc-HSA in 10-*μ*m-thick tumour sections at a spatial resolution of just above 50 *μ*m. The images could be subjected to quantitative analysis to determine the uptake of ^99m^Tc-HSA in any freely selected tumour subregion. Moreover, correlations between the uptake of ^99m^Tc-HSA and histological characteristics could be searched for by superimposing Bioscope images on histological images obtained from the same tumour sections.

Studies of intratumour heterogeneity in the uptake of macromolecules using a radiolabelled molecule as a probe require specific precautions to avoid experimental artefacts and incorrect interpretation of data. Degradation of the probe, resulting in unbound and hence freely diffusible activity, may generate data indicating that the heterogeneity is lower than the true heterogeneity. ^99m^Tc-HSA was used as a model molecule for macromolecular therapeutic agents in the present work. Intratumour heterogeneity in ^99m^Tc-HSA concentration was seen within a few minutes after the administration of the probe, and this initial heterogeneity was maintained at unchanged magnitude throughout the whole observation period of 180 min. In contrast, control experiments with Na^99m^TcO_4_ showed that the initial heterogeneity in Na^99m^TcO_4_ concentration decayed rapidly with time, resulting in a homogeneous distribution within less than an hour. Consequently, degradation of ^99m^Tc-HSA probably did not represent a serious problem in our study.

Significant intratumour heterogeneity in the uptake of ^99m^Tc-HSA was detected. The heterogeneity had two distinctly different components, one random and one radial component. In non-necrotic tissue, the uptake was lowest in the centre of the tumours and increased towards the tumour periphery. The radial distribution of ^99m^Tc-HSA was initiated within minutes after the administration of ^99m^Tc-HSA and was maintained at least until maximum uptake was attained, approximately 180 min later. Mathematical modelling has suggested that radial heterogeneity in the uptake of macromolecules in tumours could be because of radial heterogeneity in blood perfusion, histopathological characteristics, microvascular permeability and/or interstitial fluid pressure (Baxter and Jain, 1988, 1990, 1990; Jain and Baxter, 1988; Fujimori *et al*, 1989; Thomas *et al*, 1989).

The radial intratumour heterogeneity in blood perfusion and extracellular volume fraction was determined experimentally in the present work by using ^99m^Tc-labelled molecules as tracers and the Bioscope imaging system for detection. Experimental studies with the same melanoma lines of intratumour heterogeneity in the permeability of the microvasculature to Gd-labelled HSA and intratumour heterogeneity in interstitial fluid pressure have been reported previously (Bjørnæs and Rofstad, 2001; Rofstad *et al*, 2002). As discussed below, the biological factors leading to the radial heterogeneity in the uptake of ^99m^Tc-HSA can be identified from these studies.

The radial heterogeneity in the uptake of ^99m^Tc-HSA appeared within minutes after the administration of ^99m^Tc-HSA in both A-07 and R-18 tumours. The blood perfusion in these tumours showed a radial gradient similar to that in the uptake of ^99m^Tc-HSA. These two observations together strongly suggest that intratumour heterogeneity in the supply of ^99m^Tc-HSA through the microvasculature contributed significantly to the initiation of the radial gradients in ^99m^Tc-HSA uptake.

We have reported previously that our melanoma lines show an intertumour heterogeneity in the uptake of albumin that is governed primarily by intertumour differences in extracellular volume fraction (Graff *et al*, 2000). However, intratumour heterogeneity in extracellular volume fraction probably did not contribute significantly to the radial gradients in ^99m^Tc-HSA uptake, since neither A-07 nor R-18 tumours showed strong radial gradients in extracellular volume fraction.

Quantitative studies of the permeability of the microvascular wall of A-07 and R-18 tumours to Gd-labelled HSA have been reported elsewhere (Bjørnæs and Rofstad, 2001). The effective microvascular permeability constant was found to be extremely high, suggesting that the uptake of HSA in these tumours is not inhibited significantly by the microvascular wall. However, intratumour heterogeneity in the effective microvascular permeability constant was detected, but this heterogeneity was random. Consequently, intratumour heterogeneity in the permeability of the microvascular wall probably did not contri-bute significantly to the radial gradients in ^99m^Tc-HSA uptake measured here.

Measurements of the radial heterogeneity in interstitial fluid pressure (IFP) in A-07 tumours have been reported recently (Rofstad *et al*, 2002). These measurements showed that the IFP increased with increasing distance into the tumours until a plateau was reached at a depth of 0.75 mm, that is, the tumours showed a steep IFP gradient in the periphery and relatively uniform IFP values at depths beyond 0.75 mm. A depth of 0.75 mm corresponds to a normalised radius >0.8 in the tumours used here, implying that the radial gradients in ^99m^Tc-HSA uptake did not correspond to the radial gradients in IFP (Rofstad *et al*, 2002). One possible explanation is that convection may not be the most important transport mechanism for ^99m^Tc-HSA (Perl, 1975). Another possible explanation is that ^99m^Tc-HSA delivered in the tumour periphery was transported out of the tissue and into the surrounding skin because of the steep IFP gradient in the tumour periphery. In any case, the radial gradients in ^99m^Tc-HSA uptake seen here were not primarily a consequence of radial gradients in IFP.

The radial gradients in the uptake of ^99m^Tc-HSA seen shortly after the administration of ^99m^Tc-HSA was maintained for at least 180 min, that is, until maximum global uptake was reached. This observation shows that the interstitial transport of ^99m^Tc-HSA from peripheral tumour regions with high blood perfusion to central tumour regions with low blood perfusion was inefficient. A dense and well-organised extracellular matrix may have prevented efficient transport by diffusion (Hobbs *et al*, 1998; Krol *et al*, 1999), whereas convection may have caused transport from the centre to the periphery of the tumours, that is, in the opposite direction (Jain and Baxter, 1988).

Consequently, the studies discussed here collectively suggest that the tumour microvasculature and the tumour interstitium represented significant transport barriers for ^99m^Tc-HSA in our melanoma xenografts, causing radial gradients in the uptake of ^99m^Tc-HSA in non-necrotic tissue. The gradients were initiated primarily because of radial heterogeneity in the supply of ^99m^Tc-HSA through the microvasculature. This radial heterogeneity was maintained at least until maximum global uptake was attained, mainly because of the presence of significant transport barriers in the tumour interstitium preventing equalisation of the initial heterogeneity with time.

The random component of the intratumour heterogeneity in the uptake of ^99m^Tc-HSA was most likely also caused by the same transport barriers, that is, heterogeneous supply through the tumour microvasculature and inadequate transport through the tumour interstitium. This conclusion follows from the observations that spots with high ^99m^Tc-HSA uptake colocalised with spots with high vascular density and regions without significant ^99m^Tc-HSA uptake colocalised with necrotic regions, that is, regions at long distances from functional vessels.

If our melanoma xenografts are representative models of cancer in humans, the study reported here suggests that strategies for improving the delivery of macromolecular therapeutic agents to tumours should focus on increasing the tumour blood perfusion and improving the diffusion conditions in the tumour interstitium. The increased perfusion may lead to an enhancement of the total macromolecule uptake and improved diffusion conditions may lead to a more homogeneous macromolecule distribution in the tumour tissue.
